# Enhancing Early Dementia Detection in Primary Care with a Culturally Tailored Bilingual EHR Screening Tool

**DOI:** 10.1002/alz70858_105676

**Published:** 2025-12-26

**Authors:** Samantha Shah, Satpal S. Wadhwa, Gabriela Islas Huerta, Stephanie G Ovalle Eliseo, Ariadna Martinez, Blanca Campos, Keith Vossel, Michelle Bholat, Mirella Diaz‐Santos, Timothy S Chang

**Affiliations:** ^1^ David Geffen School of Medicine, University of California, Los Angeles, Los Angeles, CA, USA; ^2^ David Geffen School of Medicine at UCLA, Los Angeles, CA, USA; ^3^ University of California Los Angeles, Department of Family Medicine, Los Angeles, CA, USA; ^4^ University of California, Los Angeles, Los Angeles, CA, USA; ^5^ Movement Disorders Programs, Department of Neurology, David Geffen School of Medicine, University of California, Los Angeles, Los Angeles, CA, USA

## Abstract

**Background:**

Alzheimer's disease (AD) affects over 10% of individuals aged 65 and older, with Black and Hispanic/Latino individuals experiencing a 1.5‐2.0 times higher prevalence than white individuals. Despite these disparities, AD remains underdiagnosed, particularly in non‐white populations. Current dementia screening tools, such as the Mini‐Mental State Examination (MMSE) and Montreal Cognitive Assessment (MoCA), face challenges in time efficiency, accessibility, and cultural sensitivity. This study aimed to implement a brief dementia screening tool integrated into the electronic health record (EHR) and evaluate its impact on diagnosis, workup, and treatment in a diverse family medicine clinic in Los Angeles County.

**Method:**

The dementia screening tool (DST), developed collaboratively by the University of California Alzheimer's Disease Centers and the California Department of Public Health, was designed to be brief (<5 minutes) and adaptable. It includes a three‐question patient questionnaire, an informant input option, and the Mini‐Cog assessment. To enhance accessibility, the tool was translated and culturally adapted for Spanish‐speaking patients. Patients aged 60+ completed the DST before their annual wellness visits, with results integrated into the EHR. This pre‐post intervention study compared patients aged 60+ without a prior dementia diagnosis during the pre‐intervention (February 2016–August 2022) and post‐intervention (September 2022–June 2023) periods. Outcomes included new dementia diagnoses, medications, specialty referrals, labs, and imaging.

**Result:**

The DST was implemented, screening 996 patients in 10 months. Screening led to 35 specialty care referrals and 15 new dementia diagnoses. New diagnoses increased from 4.17% pre‐DST to 4.80% post‐DST all (OR 2.10, *p* = 0.02) and 6.43% among those screening positive (OR 2.57, *p* = 0.04). Dementia medication prescriptions rose from 2.93% pre‐DST to 4.94% in the post‐DST all group, reaching 6.87% among those who screened positive. Specialty referrals were more frequent post‐DST all (9.13%) and even higher among those screening positive (14.16%). Post‐DST, all secondary outcomes significantly improved, including increased use of diagnostic labs and imaging studies.

**Conclusion:**

Integrating a brief, culturally sensitive DST into primary care significantly improved dementia diagnosis rates, workup, referrals, and treatment. These findings highlight the potential for broader implementation of the DST to enhance dementia care and address health disparities in diverse populations.

